# Association of internet-integrated palliative care pathway assignment with recorded quality-of-life and symptom-burden scores in patients with terminal cancer: a single-center retrospective propensity score-matched cohort study

**DOI:** 10.3389/fmed.2026.1904181

**Published:** 2026-08-19

**Authors:** Jie Xin, Ning Li, Jumei Sun, Xiaofeng Wu, Chunhui Li

**Affiliations:** 1 The People’s Hospital of Feicheng, Feicheng, Shandong, China; 2 Shenzhen Mental Health Center/Shenzhen Kangning Hospital, Shenzhen, China; 3 Feicheng Center for Disease Control and Prevention, Feicheng City, Shandong, China

**Keywords:** care-pathway assignment, palliative care, propensity score matching, quality of life, recorded questionnaire scores, symptom burden, terminal cancer

## Abstract

**Background:**

Internet-supported models may extend palliative care beyond scheduled hospital encounters, but the validity of comparisons from routine terminal-cancer care depends on clear care pathway assignment and outcome ascertainment.

**Objective:**

This study aimed to evaluate whether initial recorded assignment to an internet-integrated palliative care pathway was associated with recorded global quality-of-life and symptom-burden scores compared with conventional care.

**Methods:**

This single-center retrospective cohort screened 268 potentially eligible patients in 2023. The operational exposure was the archived group field at pathway entry (internet or conventional), analyzed according to initial recorded assignment; actual platform use, crossover, discontinuation, contact frequency, and care intensity were not verifiable. Eligibility was defined at baseline, and 224 patients entered the candidate cohort. The primary composite distance match produced 85 pairs. Recorded EORTC QLQ-C15-PAL global quality-of-life and ESAS total symptom-burden scores were analyzed with linear mixed-effects models including matched-pair and patient random intercepts. Data validation included duplicate-identifier, exposure-code, date, range, and cross-file consistency checks. Missingness analyses and a conventional 1:1 nearest-neighbor propensity-score match were used to assess robustness.

**Results:**

The primary matched cohort included 170 patients, and all absolute post-matching standardized mean differences (SMDs) were below 0.10 (maximum, 0.092). At 4 and 8 weeks, recorded global quality-of-life scores were higher by 10.88 points (95% CI, 8.71–13.05) and 16.75 points (95% CI, 14.55–18.96), respectively, and recorded total symptom-burden scores were lower by 9.94 points (95% CI, 8.22–11.65) and 17.78 points (95% CI, 16.04–19.52), respectively (all *p* < 0.001). Conventional nearest-neighbor matching yielded 88 pairs and are directionally consistent, slightly attenuated contrasts, although covariate balance was weaker (maximum absolute SMD, 0.163).

**Conclusion:**

Initial recorded assignment to the internet-integrated care pathway was associated with more favorable recorded questionnaire-score trajectories in the selected matched common-support population. Unverified administration metadata, possible differential measurement error, non-random assignment, incomplete implementation data, and inability to reconstruct visit-specific mortality preclude causal attribution to the internet component.

## 1 Introduction

Patients with terminal cancer often experience a high burden of multidimensional symptoms, including pain, fatigue, anorexia, psychological distress, sleep disturbances, and declining physical function. These symptoms frequently coexist with progressive functional deterioration, complex medication needs, and increasing dependence on family caregivers. As disease-directed treatment becomes limited or no longer appropriate, the goals of care increasingly shift toward symptom relief, preservation of quality of life, communication about care preferences, and support for patients and families. Early and integrated palliative care has been associated with improved patient-reported outcomes in advanced cancer populations, but maintaining continuity of supportive care remains challenging in real-world practice (1, 2).

Digital and telehealth approaches have therefore been incorporated into palliative care through remote consultations, electronic symptom reporting, education, and coordination. Randomized and observational studies support the feasibility of remote palliative care, and electronic patient-reported outcome systems can improve symptom communication (3–6). However, systematic reviews also identify digital-access barriers, caregiver dependence, workflow constraints, and heterogeneous intervention effects (7–11). Telepalliative care should not be presumed superior merely because it is technologically enabled.

Conventional palliative care is often delivered through outpatient visits, telephone follow-up, emergency consultations, and hospitalization when symptoms worsen. Internet-integrated pathways may create additional opportunities for symptom submission, professional communication, education, medication coordination, and home-care referral (12–14). These functions can also increase the intensity and continuity of care, making it difficult to separate the effect of the internet interface from the effect of receiving more frequent or coordinated support.

Evidence remains mixed. A multisite trial found video-based early palliative care non-inferior to in-person care (3), and a small specialized outpatient trial found telemedicine plus standard care not inferior to standard care alone (15). Conversely, weekly specialist teleconsultations did not improve symptom burden in another randomized trial and were associated with less favorable symptom findings (16). Few routine-care studies have combined baseline-defined cohort construction, detailed matching diagnostics, longitudinal record questionnaire scores, and explicit sensitivity analyses for informative missingness. We therefore evaluated whether initial recorded assignment to an internet-integrated palliative care pathway was associated with 4- and 8-week trajectories of recorded global quality-of-life and total symptom-burden scores compared with conventional care among patients retained in a matched common-support population.

## 2 Materials and methods

### 2.1 Study design and setting

We conducted a single-center retrospective propensity score-matched cohort study using routine clinical records from the People’s Hospital of Feicheng. Both care pathways were components of clinical practice rather than interventions introduced for this study. The exposure contrast was initial recorded pathway assignment, and the reporting follows the STROBE statement (17).

### 2.2 Data source, cohort construction, and exposure assignment

**Figure 1:**
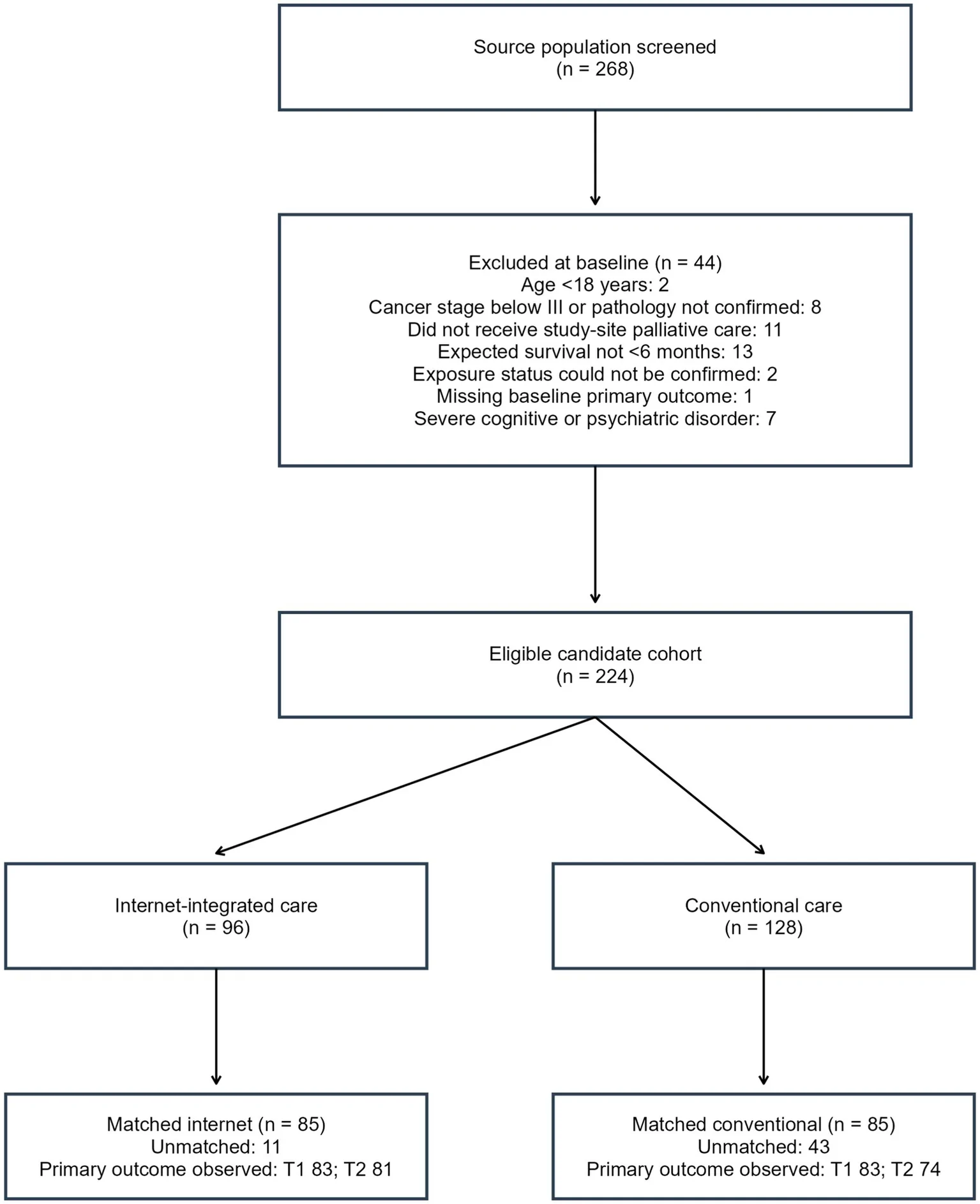
Patient flow from the 268-recorded source population through baseline exclusions, candidate-cohort construction, initial recorded pathway assignment, propensity score matching, and observed principal-score follow-up. Eligibility did not require later follow-up completion.

The source screening file contained 268 potentially eligible patients identified from oncology, palliative-care referral, and related institutional records between 1 January and 31 December 2023. Records were linked across the electronic medical record, the institutional palliative care database, and the archived group field. This field contained two values—‘internet’ and ‘conventional’—and represented initial recorded enrollment in the assigned care pathway at the index date, not verified subsequent platform use. The derived internet_flag variable exactly reproduced group assignment. Eligibility was assessed from information available at cohort entry, and later T1/T2 completion was not required. Of 268 screened patients, 44 were excluded for one mutually exclusive baseline reason: age below 18 years (*n* = 2), cancer stage below III or unconfirmed pathology (*n* = 8), expected survival not documented as below 6 months (*n* = 13), no study-site palliative care (*n* = 11), severe cognitive or psychiatric disorder precluding assessment (*n* = 7), missing baseline primary outcome (*n* = 1), or inability to confirm exposure status (*n* = 2). The remaining 224 patients formed the candidate cohort before matching (Figure 1).

The index date was the recorded date of initial care-pathway assignment and T0 assessment. Assignment and T0 shared a calendar date, but within-day ordering could not be reconstructed. Baseline QLQ-C15-PAL and ESAS values were therefore assessed in balance diagnostics and baseline-adjusted outcome models but were not included in the primary propensity score. No source-verifiable field captured actual platform engagement, crossover, discontinuation, or switching; all analyses followed initial recorded assignment.

### 2.3 Participants and sample size

Patients were eligible if they were aged 18 years or older; had pathologically confirmed stage III or IV cancer; were documented by the attending physician or palliative care team as having an expected survival below 6 months; received study-site palliative care during the study period; had an available T0 global quality-of-life score; and had a confirmable care pathway. Stage III disease was eligible only when routine records indicated incurable disease or no further disease-directed treatment together with the limited-prognosis determination. Early-stage cancers were outside the hospital program’s intended terminal-cancer population and were not eligible. The archived screening extract retained the clinician’s prognosis determination but not a named prognostic instrument or its individual components; therefore, a standardized prognostic score could not be reconstructed.

Patients with severe cognitive or psychiatric disorders precluding the archived assessment, missing T0 primary outcome, or unconfirmable exposure status were excluded. No patient was excluded solely because T1 or T2 data were missing. Because this was a retrospective cohort of all baseline-eligible records during the study period, no prospective sample-size or power calculation was performed. The final analytical size was determined by the final locked matching procedure.

### 2.4 Exposure and control conditions

Care pathway assignment was non-random and occurred in routine practice. Available records indicate that initial assignments could reflect clinician recommendation, patient or caregiver preference, digital access, caregiver support, service availability, and feasibility of remote follow-up. Because actual use and crossover were not verifiable, the exposure contrast estimates the association with assignment to a care pathway rather than receipt of a quantified internet intervention. These factors create potential selection bias even after measured-confounding adjustment.

The internet-integrated pathway combines conventional palliative care with enrollment in a pathway offering platform-supported communication and symptom submission, online professional support, patient/caregiver education, and coordination of medication delivery, nursing appointments, home-care contacts, and referral when clinically indicated. Symptom submissions could prompt clinical review and follow-up according to routine practice; however, no standardized source-verifiable alert threshold or escalation protocol was retained. Patient-level utilization counts, contact frequency, response times, staffing, discontinuation, and care intensity were also unavailable. The pathway was therefore analyzed as a complex assignment, and no observed association was attributed to an individual platform function.

The conventional pathway included routine outpatient review, telephone follow-up, face-to-face or inpatient assessment when clinically necessary, health education, and usual symptom management. It did not include initial enrollment in the structured internet pathway. Because both pathways could include telephone contact and clinically indicated escalation, and comparative contact intensity was unavailable, the contrast is between initial routine-care pathway assignments rather than between internet use and no contact.

### 2.5 Recorded questionnaire scores and assessment time points

The primary outcome was the recorded global quality-of-life score identified in the hospital database as the EORTC QLQ-C15-PAL, on a 0–100 scale with higher scores indicating better quality of life (18). Supportive recorded QLQ-C15-PAL scores were physical functioning, pain, and fatigue, using the published scale direction (18). The key secondary outcome was the recorded nine-item ESAS total score, ranging from 0 to 90, with higher scores indicating greater symptom burden (19).

The archive did not retain questionnaire language/form-version identifiers, item-level source forms, administration mode, or self- versus proxy-completion indicators, and it did not distinguish the original ESAS layout from ESAS-r. Consequently, standardized administration across groups and visits and independent rescoring could not be demonstrated. We therefore use the term recorded questionnaire scores and regard possible differential measurement error as a major limitation. A locked validation audit found no duplicate candidate or patient-visit identifiers, invalid group codes, chronological or stored-day inconsistencies, out-of-range QLQ-C15-PAL or ESAS total values, or discrepancies against the archived candidate workbook; item-level scoring checks were not possible because item-level forms were absent.

T0 was baseline; T1 targeted 4 weeks (prespecified window, 21–35 days); and T2 targeted 8 weeks (49–63 days). T0–T2 constituted the primary longitudinal period. T3 denoted a heterogeneous withdrawal or final assessment rather than a scheduled endpoint and was analyzed only exploratorily.

The archived variable labeled FAMCARE_total was moved out of the primary and key secondary hierarchy. Its version, respondent (patient or caregiver), continuity of respondent across visits, administration mode, and scoring workflow could not be reliably verified (20). Moreover, baseline values were missing for 71 of 170 matched patients although T1 and T2 fields were complete. It was therefore reported descriptively in the Supplementary Material without confirmatory modeling and was not termed a patient-reported outcome.

### 2.6 Covariates

Baseline covariates were age, sex, education level, monthly income level, marital status, caregiver relationship, body mass index, Karnofsky Performance Status, cancer stage, diagnosis category, pain-treatment history, and complication-count category. Rare caregiver categories were combined as other/none for propensity modeling while the original categories were retained for descriptive balance assessment.

### 2.7 Propensity score matching

A logistic model estimated the probability of internet-integrated care using age, sex, education, monthly income, Karnofsky Performance Status, cancer stage, diagnosis category, pain-treatment history, complication category, marital status, and caregiver relationship. Body mass index was retained in balance diagnosis. Baseline QLQ-C15-PAL and ESAS values were not placed in the propensity model because exposure and T0 had the same recorded date and within-day ordering was indeterminate; both were included in balance diagnostics and outcome-model sensitivity analyses.

One-to-one matching without replacement was restricted to common support and a caliper of 0.20 standard deviations of the logit propensity score (caliper = 0.1133) (21–23). Within eligible caliper edges, the primary locked composite-distance algorithm selected pairs in ascending composite distance. Composite distance equals the absolute logit-propensity difference plus 0.20 times a standardized auxiliary covariate distance. The auxiliary distance included standardized age and Karnofsky Performance Status plus indicator-coded sex, education, monthly income, marital status, collapsed caregiver relationship, cancer stage, diagnosis category, pain-treatment history, and complication category. The three-level pain-treatment indicator block was weighted 0.5 before calculation to limit its influence after dummy expansion. Body mass index and baseline QLQ-C15-PAL/ESAS scores did not enter the auxiliary distance. The 0.20 and 0.5 weights were pragmatic rather than derived from a standard matching estimator. Ties were resolved by the logit distance and patient identifier. Balance was acceptable when every absolute SMD was below 0.10. Because this distance is non-standard, a conventional nearest-neighbor propensity-score sensitivity analysis was added as specified in Section 2.9.

### 2.8 Missing data and withdrawal

Observed and missing values were tabulated by group, outcome, and visit. Available T1/T2 missing-reason fields distinguished temporary hospitalization, patient fatigue, missed remote assessment, and unknown reason. Completion was defined using QLQ-C15-PAL global and ESAS total values only; the exploratory FAMCARE field did not determine completion.

The mixed models use all available repeated outcomes under a missing-at-random assumption. Informative missingness was assessed with stabilized inverse-probability-of-observation weights estimated separately for the two principal outcomes. The observation models included group, visit, age, sex, Karnofsky Performance Status, stage, diagnosis, marital status, caregiver relationship, complications, baseline outcome, previous outcome, and previous-visit completion. Weights were truncated in the 1st and 99th percentiles. A differential pattern-mixture stress test imputed missing conventional-care values at model-predicted values and shifted missing internet-care values in the unfavorable direction by 5 and 10 points for global quality of life and by 3 and 6 points for ESAS total.

Recorded T3 reason categories included death, loss to follow-up, stopped intervention, transfer, other, and not recorded. A dedicated death-date field was not retained, so deaths before T1 and T2 could not be separated from other missingness and visit-specific mortality could not be reconstructed. Questionnaire scores after death were regarded as undefined and were neither assigned zero nor mechanically imputed. An observed score necessarily indicates that the patient was alive and observable at that assessment, but vital status could not be classified for patients without a score. Follow-up contrasts therefore describe differences among patients represented by the observed longitudinal score process under the model assumptions; they are not survivor-average or death-adjusted causal estimands.

### 2.9 Statistical analysis

Continuous variables are summarized as mean±standard deviation and categorical variables as *n* (%). The primary T0–T2 models were linear mixed-effects models with fixed effects for initially assigned groups, categorical time, and group-by-time interaction; random intercepts were nested for matched pairs and patients. Estimated marginal means and internet-assignment-minus-conventional-assignment contrasts with 95% confidence intervals are reported at each time point. The target estimand is the average difference in recorded questionnaire scores associated with initial pathway assignment among patients retained in the matched common-support population, rather than an effect for the full eligible cohort. Supportive outcomes used the same model, with Benjamini–Hochberg false-discovery-rate adjustment across T1 and T2 contrasts.

Sensitivity analyses included adjustment for age, sex, Karnofsky Performance Status, diagnosis, marital status, and caregiver relationship; explicit baseline-outcome adjustment at T1/T2; a patient-only random-intercept model; inverse-probability-of-observation weighting; differential pattern-mixture scenarios; restriction to T1/T2 target windows; and exploratory addition of T3. In addition, conventional 1:1 nearest-neighbor propensity-score matching used the same propensity model, common support, a 0.20-standard-deviation logit-propensity caliper, no replacement, no auxiliary covariate-distance term, deterministic ordering of internet-assigned patients from highest to lowest logit propensity, nearest available conventional patient, and patient-identifier tie-breaking. The principal QLQ-C15-PAL global and ESAS total mixed models were repeated in this sensitivity cohort. Two-sided *p* < 0.05 was used for the principal outcomes. R version 4.5.2 was used with nlme 3.1–168 and the packages listed in the archived analysis code.

### 2.10 Ethics approval

The study protocol was reviewed and approved by the Ethics Committee of the People’s Hospital of Feicheng (approval no. 2023102). The requirement for written informed consent was waived because this was a retrospective study using de-identified clinical data.

## 3 Results

### 3.1 Cohort construction and propensity score matching

Among 268 screened patients, 44 were excluded at baseline, and 224 entered the candidate cohort (96 internet-integrated care and 128 conventional care). Common support contained all 96 internet-care patients and 116 conventional-care patients. Matching yielded 85 pairs (170 patients); 11 internet-care and 43 conventional-care candidates were unmatched (Figure 1).

### 3.2 Baseline characteristics and covariate balance

**Figure 2:**
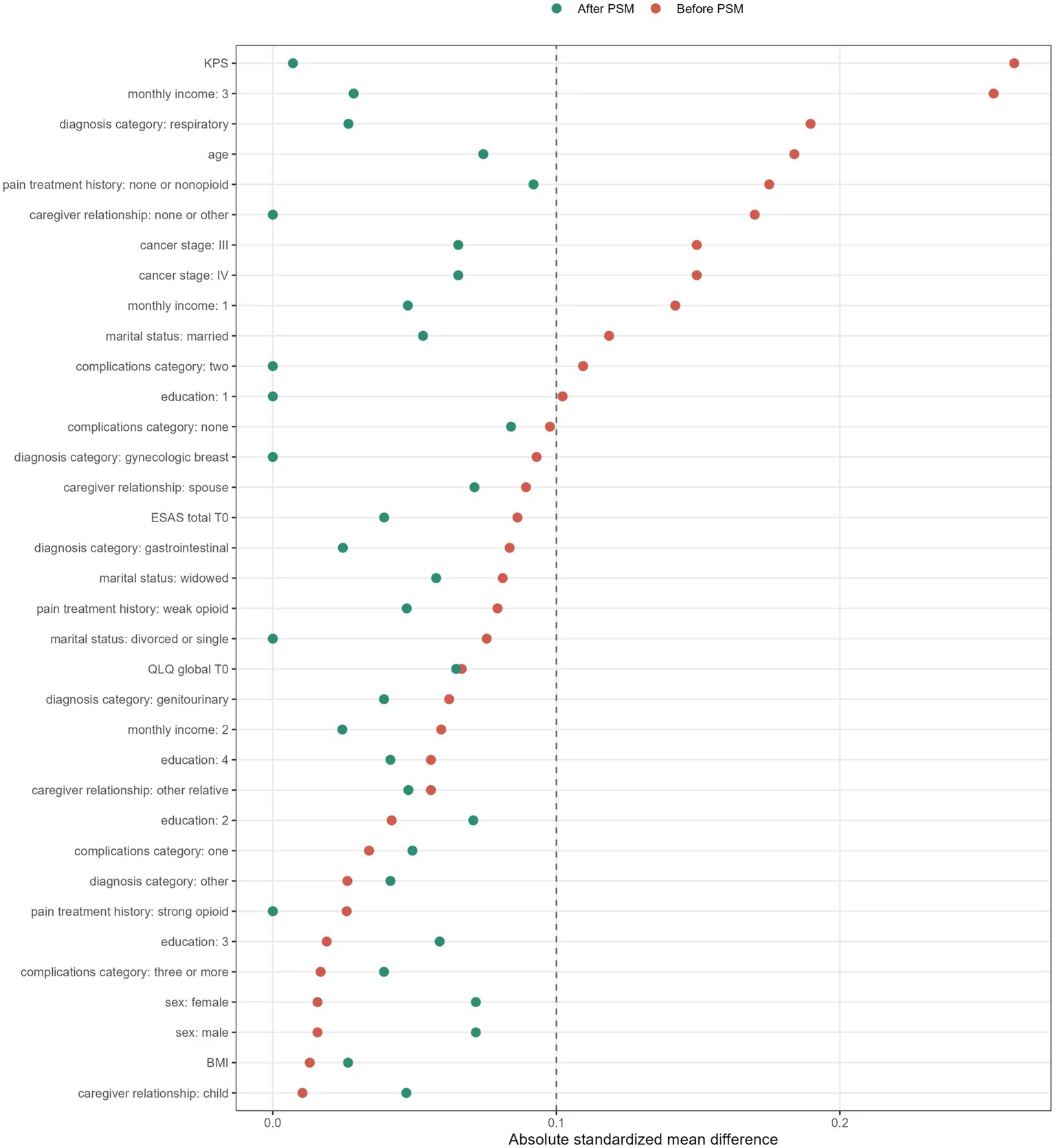
Absolute standardized mean differences before and after propensity score matching. The dashed vertical line denotes 0.10; all post-matching values were below this threshold.

The maximum absolute SMD was 0.262 before matching and 0.092 after the primary composite-distance match. All measured baseline covariates, including marital status, caregiver relationship, body mass index, T0 global quality of life, and T0 ESAS total, were below 0.10 after matching (Table 1; Figure 2; Supplementary Table S4). The baseline internet-minus-conventional differences were −0.40 points for recorded global quality of life and −0.20 points for recorded ESAS total. The locked data-validation audit found zero duplicate candidate or patient-visit identifiers, invalid group codes, group/internet_flag mismatches, date/day inconsistencies, out-of-range principal scores, or archived-source discrepancies.

**Table 1:** Baseline characteristics of the matched cohort.

Characteristic	Conventional care (*n* = 85)	Internet-integrated care (*n* = 85)	Absolute SMD
Age, years	67.27 ± 9.62	66.56 ± 9.39	0.074
BMI, kg/m^2^	21.28 ± 3.03	21.20 ± 2.91	0.027
KPS	59.35 ± 8.37	59.29 ± 8.17	0.007
QLQ-C15-PAL global score at T0	67.67 ± 6.04	67.28 ± 6.26	0.065
ESAS total at T0	32.35 ± 5.11	32.16 ± 4.96	0.039
Sex: female	34 (40.0)	37 (43.5)	0.072
Sex: male	51 (60.0)	48 (56.5)	0.072
Education level 1	20 (23.5)	20 (23.5)	0.000
Education level 2	42 (49.4)	39 (45.9)	0.071
Education level 3	16 (18.8)	18 (21.2)	0.059
Education level 4	7 (8.2)	8 (9.4)	0.041
Monthly income level 1	37 (43.5)	35 (41.2)	0.048
Monthly income level 2	30 (35.3)	31 (36.5)	0.025
Monthly income level 3	18 (21.2)	19 (22.4)	0.029
Marital status: divorced or single	5 (5.9)	5 (5.9)	0.000
Marital status: married	63 (74.1)	61 (71.8)	0.053
Marital status: widowed	17 (20.0)	19 (22.4)	0.058
Caregiver relationship: child	40 (47.1)	42 (49.4)	0.047
Caregiver relationship: none or other	1 (1.2)	1 (1.2)	0.000
Caregiver relationship: other relative	5 (5.9)	6 (7.1)	0.048
Caregiver relationship: spouse	39 (45.9)	36 (42.4)	0.071
Cancer stage: III	14 (16.5)	12 (14.1)	0.065
Cancer stage: IV	71 (83.5)	73 (85.9)	0.065
Diagnosis category: gastrointestinal	29 (34.1)	30 (35.3)	0.025
Diagnosis category: genitourinary	9 (10.6)	8 (9.4)	0.039
Diagnosis category: gynecologic breast	17 (20.0)	17 (20.0)	0.000
Diagnosis category: other	7 (8.2)	8 (9.4)	0.041
Diagnosis category: respiratory	23 (27.1)	22 (25.9)	0.027
Pain treatment history: none or non-opioid	5 (5.9)	7 (8.2)	0.092
Pain treatment history: strong opioid	40 (47.1)	40 (47.1)	0.000
Pain treatment history: weak opioid	40 (47.1)	38 (44.7)	0.047
Complication category: none	18 (21.2)	21 (24.7)	0.084
Complication category: one	31 (36.5)	29 (34.1)	0.049
Complication category: three or more	9 (10.6)	8 (9.4)	0.039
Complication category: two	27 (31.8)	27 (31.8)	0.000

Continuous variables are mean ± SD and categorical variables are *n* (%). Education and income thresholds were not retained in the archived analytical extract and are therefore shown as coded levels. All absolute SMDs were <0.10.

### 3.3 Missing data, follow-up completeness, and withdrawal

At T1, primary and key secondary outcomes were observed for 83 of 85 patients in each group. At T2, they were observed for 74 of 85 conventional-care patients and 81 of 85 internet-care patients. Overall, 151 of 170 patients had complete QLQ-C15-PAL global and ESAS total values at T0–T2 (72 conventional and 79 internet). Patients with complete follow-up had mean baseline Karnofsky scores of 59.4 versus 58.7 among those with incomplete follow-up; incomplete follow-up remained more frequent in conventional care (Supplementary Tables S1–S3).

T1 fell within 21–35 days for 83 conventional and 84 internet-assigned records; T2 fell within 49–63 days for 79 and 81 records, respectively. At T3/final assessment, death was the recorded reason for 29 conventional-care and 25 internet-assigned patients. Because exact death dates were unavailable, the number of deaths before each scheduled T1/T2 assessment could not be reconstructed or separated from other missingness (Supplementary Table S2).

### 3.4 Primary outcome: recorded global quality-of-life score

**Figure 3:**
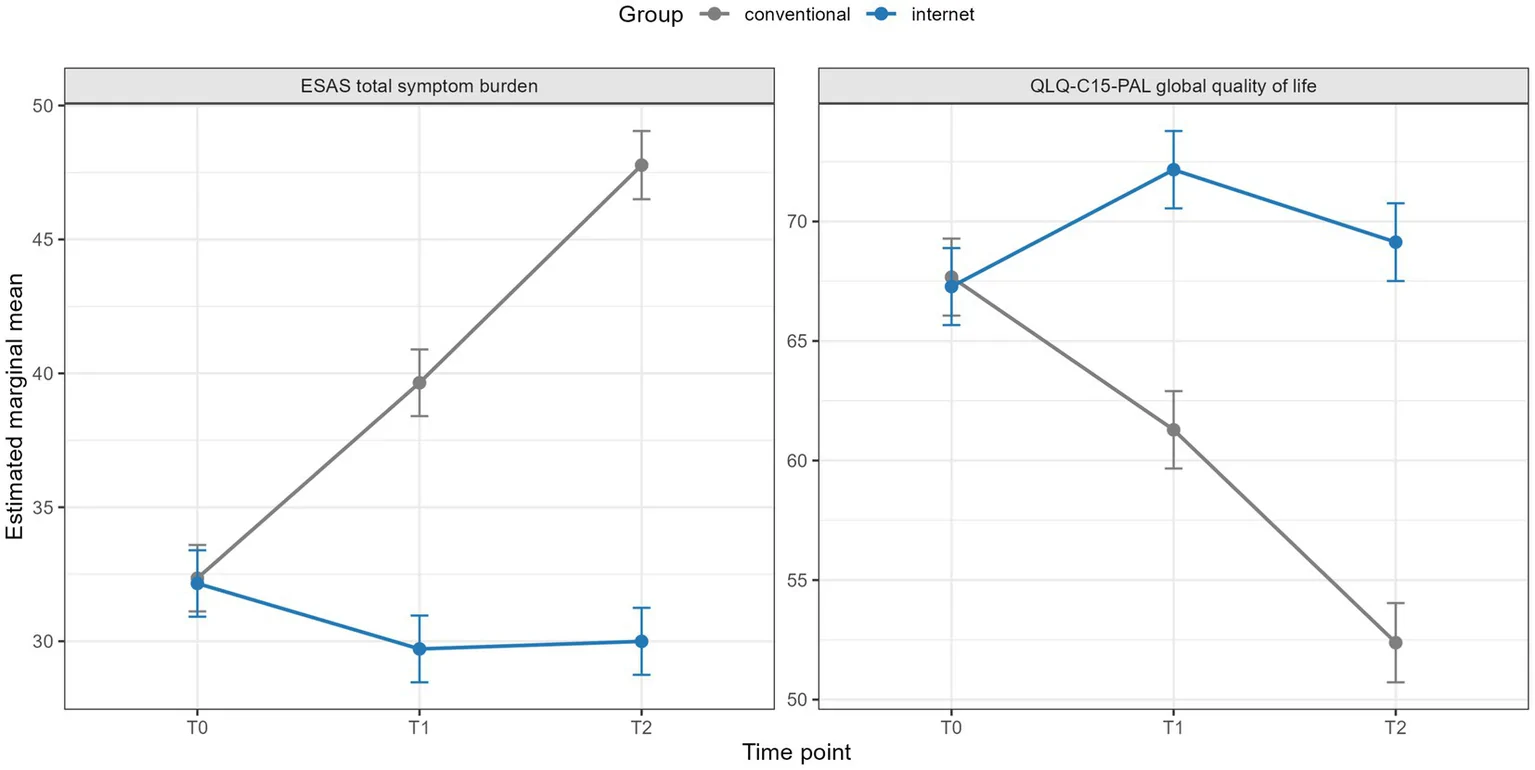
Estimated marginal means and 95% confidence intervals for recorded ESAS total symptom burden and QLQ-C15-PAL global quality-of-life scores at T0, T1, and T2. Models include matched-pair and patient random intercepts; groups represent initial recorded pathway assignment.

The group-by-time interaction for the recorded global quality-of-life score was significant (*F* = 192.34; df = 2,317; *p* < 0.001). Observed means in conventional care were 67.67 ± 6.04 at T0, 61.29 ± 7.78 at T1, and 52.28 ± 7.88 at T2; corresponding internet-assigned means were 67.28 ± 6.26, 72.18 ± 9.09, and 69.39 ± 8.11. The model-based internet-assignment-minus-conventional-assignment difference was −0.40 points (95% CI, −2.55 to 1.76; *p* = 0.718) at T0, 10.88 (95% CI, 8.71–13.05; *p* < 0.001) at T1, and 16.75 (95% CI, 14.55–18.96; *p* < 0.001) at T2 (Table 2; Figure 3).

**Table 2:** Primary outcome: recorded QLQ-C15-PAL global quality-of-life score.

Time	Conventional: observed mean ± SD; EMM (95% CI)	Internet: observed mean ± SD; EMM (95% CI)	Difference (95% CI)	*p*
T0	*n* = 85; 67.67 ± 6.04; EMM 67.67 (66.06–69.28)	*n* = 85; 67.28 ± 6.26; EMM 67.28 (65.67–68.89)	−0.40 (−2.55 to 1.76)	0.718
T1	*n* = 83; 61.29 ± 7.78; EMM 61.29 (59.67–62.90)	*n* = 83; 72.18 ± 9.09; EMM 72.17 (70.55–73.78)	10.88 (8.71 to 13.05)	<0.001
T2	*n* = 74; 52.28 ± 7.88; EMM 52.38 (50.73–54.04)	*n* = 81; 69.39 ± 8.11; EMM 69.14 (67.51–70.76)	16.75 (14.55 to 18.96)	<0.001

Difference is initial internet-pathway assignment minus conventional-pathway assignment. Higher is better. Group×time interaction: F = 192.34, df = 2,317, *p* < 0.001.

### 3.5 Key secondary outcome: recorded ESAS total symptom-burden score

The group-by-time interaction for the recorded ESAS total score was significant (*F* = 354.76; df = 2,317; *p* < 0.001). Observed means in conventional care were 32.35 ± 5.11 at T0, 39.78 ± 5.43 at T1, and 47.67 ± 5.75 at T2; corresponding internet-assigned means were 32.16 ± 4.96, 29.70 ± 6.13, and 30.09 ± 7.41. The model-based difference was −0.20 points (95% CI, −1.91 to 1.51; *p* = 0.821) at T0, −9.94 (95% CI, −11.65 to −8.22; *p* < 0.001) at T1, and −17.78 (95% CI, −19.52 to −16.04; *p* < 0.001) at T2 (Table 3; Figure 3).

**Table 3:** Key secondary outcome: recorded ESAS total symptom burden score.

Time	Conventional: observed mean ± SD; EMM (95% CI)	Internet: observed mean ± SD; EMM (95% CI)	Difference (95% CI)	*p*
T0	*n* = 85; 32.35 ± 5.11; EMM 32.35 (31.11–33.59)	*n* = 85; 32.16 ± 4.96; EMM 32.16 (30.92–33.40)	−0.20 (−1.91 to 1.51)	0.821
T1	*n* = 83; 39.78 ± 5.43; EMM 39.65 (38.40–40.89)	*n* = 83; 29.70 ± 6.13; EMM 29.71 (28.47–30.96)	−9.94 (−11.65 to −8.22)	<0.001
T2	*n* = 74; 47.67 ± 5.75; EMM 47.77 (46.50–49.05)	*n* = 81; 30.09 ± 7.41; EMM 30.00 (28.74–31.25)	−17.78 (−19.52 to −16.04)	<0.001

Difference is initial internet-pathway assignment minus conventional-pathway assignment. Higher is worse. Group × time interaction: F = 354.76, df = 2,317, *p* < 0.001.

### 3.6 Supportive outcomes

At T1 and T2, physical-function scores were higher, and pain and fatigue scores were lower in the initially internet-assigned group. All six contrasts remained significant after false-discovery-rate correction (Table 4; Supplementary Figure S2). These analyses were supportive and not used as the principal basis for inference.

**Table 4:** Supportive recorded QLQ-C15-PAL outcome score contrasts.

Outcome	Time	Difference (95% CI)	Nominal *p*	FDR-adjusted *p*
Physical functioning	T1	11.66 (8.39 to 14.94)	<0.001	<0.001
Physical functioning	T2	17.24 (13.86 to 20.62)	<0.001	<0.001
Pain	T1	−10.24 (−12.78 to −7.70)	<0.001	<0.001
Pain	T2	−12.77 (−15.40 to −10.14)	<0.001	<0.001
Fatigue	T1	−7.65 (−10.21 to −5.09)	<0.001	<0.001
Fatigue	T2	−8.61 (−11.26 to −5.95)	<0.001	<0.001

Difference is initial internet-pathway assignment minus conventional-pathway assignment. Higher physical function scores are better; higher pain and fatigue scores are worse.

### 3.7 Sensitivity and exploratory analyses

The principal effect directions were unchanged after covariate adjustment, explicit baseline-outcome adjustment, removal of the matched-pair random intercept, inverse-probability-of-observation weighting, and restriction to the prespecified T1/T2 windows (Supplementary Tables S6–S11). In the weighted model, T2 differences were 16.75 points for recorded global quality of life and −17.76 points for recorded ESAS total. Under the severe differential pattern-mixture scenario, the T2 differences remained 16.62 points for recorded global quality of life and −17.31 points for recorded ESAS total. Conventional nearest-neighbor propensity-score matching yielded 88 pairs. Its T1/T2 contrasts were 10.62 (95% CI, 8.48–12.76) and 15.70 (95% CI, 13.53–17.87) for recorded global quality of life and −9.14 (95% CI, −10.73 to −7.54) and −17.25 (95% CI, −18.87 to −15.63) for recorded ESAS total (all *p* < 0.001). These estimates were directionally consistent and slightly attenuated, but the balance was weaker than in the primary match (maximum absolute SMD, 0.163; Supplementary Table S15). These sensitivity analyses do not eliminate residual confounding or informative dropout.

The recorded FAMCARE field was summarized only descriptively. At T0, it was observed for 48 conventional and 51 internet-care patients; all 85 per group had recorded T1/T2 values. Because version and respondent continuity could not be verified, no confirmatory comparison was made (Supplementary Table S12). T3 results were directionally similar but are subject to heterogeneous timing and survivor selection and remain exploratory (Supplementary Table S14; Supplementary Figure S3).

## 4 Discussion

In this baseline-defined retrospective cohort, initial recorded assignment to the internet-integrated palliative care pathway was associated with more favorable 4- and 8-week recorded global quality-of-life and total symptom-burden scores than conventional assignment among patients retained in the primary matched common-support population. The main composite-distance match achieved all absolute SMDs below 0.10. A conventional nearest-neighbor sensitivity analysis produced directionally consistent but slightly attenuated estimates with weaker balance. The findings concern pathway assignment and recorded scores; they do not identify the effect of actual platform use or establish causality.

The results align partly with studies showing that remote palliative care can maintain access and patient-centered outcomes. Greer et al. found video-based early palliative care non-inferior to in-person care in advanced lung cancer (3), while Bernhardt et al. reported non-inferiority of telemedicine plus specialized outpatient palliative care in a small trial (15). Electronic symptom monitoring can also facilitate communication and clinical response (4–6). However, our effect estimates are larger than those in many trials, and the conventional-care group deteriorated markedly. This contrast heightens, rather than removes, concerns about selection, differential care intensity, and documentation processes.

Evidence is not uniformly favorable. Hoek and colleagues found that adding weekly specialist teleconsultations did not improve patient-experienced symptom burden and observed less favorable symptom findings in the intervention group (16). Reviews emphasize heterogeneity in technology, clinical integration, patient frailty, caregiver involvement, and implementation barriers (7–9). Thus, the present association may reflect a package of continuity, coordination, caregiver engagement, and additional professional contact rather than an isolated internet effect. Symptom submissions could prompt clinical review and follow-up according to routine practice; however, no standardized source-verifiable alert threshold or escalation protocol was retained. Utilization, response-time, crossover, and care-intensity data were also unavailable, so these mechanisms cannot be tested.

The 10.88- and 16.75-point global quality-of-life differences are large relative to the conventional 10-point heuristic sometimes applied to EORTC scales. Nevertheless, QLQ-C15-PAL minimal important differences vary by domain and clinical context, and prior studies did not establish a single universal threshold for the global scale (24). Similarly, the ESAS total differences exceed published 3- to 6-point within-patient thresholds for improvement or deterioration, but those thresholds were derived for change within individuals, not matched between-pathway contrasts (25). The estimates therefore suggest potential clinical relevance, but direct threshold equivalence should be interpreted cautiously.

Missingness and truncation by death remain important. Primary/key-secondary recorded-score completion was 89% overall but lower in conventional care. Observation weighting and differential pattern-mixture stress tests were stable, and no post-death score was set to zero. An observed score establishes that the patient was alive and observable at that assessment, but the missing records could not be classified by vital status. The models therefore estimate recorded outcomes among patients represented by the observed longitudinal process under unverifiable assumptions. Because exact death dates were absent, deaths before T1/T2 could not be separated, visit-specific mortality could not be modeled, and neither a survivor-average nor a death-adjusted estimand could be estimated. Informative dropout related to deterioration, transfer, or caregiver capacity cannot be excluded.

The recorded FAMCARE field was not treated as a principal patient-reported outcome. Baseline missingness followed by complete follow-up fields, combined with absent versions and respondent metadata, suggests a different documentation workflow or later structured capture. Descriptive differences may be hypothesis-generating, but neither patient attribution nor longitudinal respondent continuity can be assumed.

Strengths include baseline-only eligibility, accounting for pair and patient correlation, comprehensive balance diagnostics, locked duplicate, range, coding, and cross-file consistency checks, observed means alongside model estimates, and multiple missingness, timing, and matching sensitivity analyses. Major limitations include the single-center retrospective design; non-random initial assignment; lack of verified actual platform use, crossover, discontinuation, contact frequency, response time, and care intensity; residual confounding and selection into the matched overlap population; uncertain within-day ordering; absent questionnaire version, language, item-level, administration-mode, and self/proxy metadata; possible differential measurement error; and unavailable exact death dates. The conventional nearest-neighbor sensitivity analysis also left several SMDs above 0.10. Generalizability to the full eligible cohort or other settings is uncertain. Prospective studies should verify exposure fidelity, standardize outcome administration, and prespecify the treatment of death and informative missingness.

## 5 Conclusion

Initial recorded assignment to an internet-integrated palliative care pathway was associated with more favorable recorded global quality-of-life and symptom-burden scores at 4 and 8 weeks in a selected matched common-support population. Directionally consistent nearest-neighbor and missingness sensitivity analyses support the association but do not overcome residual imbalances, unverified measurement, exposure-fidelity limitations, or unavailable visit-specific mortality. Causal effectiveness of the internet component cannot be inferred.

## Data Availability

The original contributions presented in the study are included in the article/Supplementary material, further inquiries can be directed to the corresponding author.
