# Supplementary material for: Association of internet-integrated palliative care pathway assignment with recorded quality-of-life and symptom-burden scores in patients with terminal cancer: a single-center retrospective propensity score-matched cohort study
Source: Front Med (Lausanne). 2026 Aug 19;13:1904181. doi: 10.3389/fmed.2026.1904181 (PMC13533691; doi:10.3389/fmed.2026.1904181)
Supplement: Supplementary file 1 [file Data_Sheet_1.PDF]

Supplementary Material

Supplementary Table S1. Missing data by outcome, group, and time point.

Outcome	Time	Group	Observed n	Missing n
QLQ_global	T0	conventional	85	0
QLQ_global	T1	conventional	83	2
QLQ_global	T2	conventional	74	11
QLQ_global	T0	internet	85	0
QLQ_global	T1	internet	83	2
QLQ_global	T2	internet	81	4
ESAS_total	T0	conventional	85	0
ESAS_total	T1	conventional	83	2
ESAS_total	T2	conventional	74	11
ESAS_total	T0	internet	85	0
ESAS_total	T1	internet	83	2
ESAS_total	T2	internet	81	4
QLQ_physical	T0	conventional	85	0
QLQ_physical	T1	conventional	83	2
QLQ_physical	T2	conventional	74	11
QLQ_physical	T0	internet	85	0
QLQ_physical	T1	internet	83	2
QLQ_physical	T2	internet	81	4
QLQ_pain	T0	conventional	85	0
QLQ_pain	T1	conventional	83	2
QLQ_pain	T2	conventional	74	11
QLQ_pain	T0	internet	85	0
QLQ_pain	T1	internet	83	2
QLQ_pain	T2	internet	81	4
QLQ_fatigue	T0	conventional	85	0
QLQ_fatigue	T1	conventional	83	2
QLQ_fatigue	T2	conventional	74	11
QLQ_fatigue	T0	internet	85	0
QLQ_fatigue	T1	internet	83	2
QLQ_fatigue	T2	internet	81	4
FAMCARE_total	T0	conventional	48	37
FAMCARE_total	T1	conventional	85	0
FAMCARE_total	T2	conventional	85	0
FAMCARE_total	T0	internet	51	34
FAMCARE_total	T1	internet	85	0
FAMCARE_total	T2	internet	85	0

Note. FAMCARE_total is an exploratory recorded care-satisfaction field and does not define primary/key-secondary follow-up completion.

Supplementary Table S2. Recorded T3/final-assessment reasons.

Group	Recorded reason	n	% of matched group
conventional	death	29	34.1
conventional	lost to followup	15	17.6
conventional	not recorded	8	9.4
conventional	other	7	8.2
conventional	stopped intervention	17	20.0
conventional	transfer	9	10.6
internet	death	25	29.4
internet	lost to followup	19	22.4
internet	not recorded	15	17.6
internet	other	9	10.6
internet	stopped intervention	10	11.8
internet	transfer	7	8.2

Note. Exact death dates were not reliably available. T3 dates were not treated as death dates; deaths before T1/T2 could not be reconstructed, and post-death questionnaire scores were not assigned zero or imputed.

Supplementary Table S3. Baseline characteristics by primary/key-secondary follow-up completeness.

Characteristic	Complete T0–T2 (n=151)	Incomplete T0–T2 (n=19)	Absolute SMD
Age, years	66.66±9.82	68.95±6.01	0.281
BMI, kg/m²	21.21±3.01	21.44±2.61	0.082
KPS	59.40±8.20	58.68±8.79	0.085
QLQ-C15-PAL global at T0	67.54±6.23	66.94±5.44	0.103
ESAS total at T0	32.33±5.12	31.68±4.23	0.138
sex: female	65 (43.0)	6 (31.6)	0.239
sex: male	86 (57.0)	13 (68.4)	0.239
education: 1	35 (23.2)	5 (26.3)	0.073
education: 2	75 (49.7)	6 (31.6)	0.375
education: 3	31 (20.5)	3 (15.8)	0.123
education: 4	10 (6.6)	5 (26.3)	0.551
monthly income: 1	65 (43.0)	7 (36.8)	0.127
monthly income: 2	55 (36.4)	6 (31.6)	0.102
monthly income: 3	31 (20.5)	6 (31.6)	0.254
marital status: divorced or single	9 (6.0)	1 (5.3)	0.030
marital status: married	109 (72.2)	15 (78.9)	0.158
marital status: widowed	33 (21.9)	3 (15.8)	0.156
caregiver relationship: child	73 (48.3)	9 (47.4)	0.020
caregiver relationship: none or other	2 (1.3)	0 (0.0)	0.164
caregiver relationship: other relative	11 (7.3)	0 (0.0)	0.396
caregiver relationship: spouse	65 (43.0)	10 (52.6)	0.193
cancer stage: III	21 (13.9)	5 (26.3)	0.313
cancer stage: IV	130 (86.1)	14 (73.7)	0.313
diagnosis category: gastrointestinal	51 (33.8)	8 (42.1)	0.172
diagnosis category: genitourinary	16 (10.6)	1 (5.3)	0.198
diagnosis category: gynecologic breast	31 (20.5)	3 (15.8)	0.123
diagnosis category: other	14 (9.3)	1 (5.3)	0.155
diagnosis category: respiratory	39 (25.8)	6 (31.6)	0.127
pain treatment history: none or nonopioid	11 (7.3)	1 (5.3)	0.083
pain treatment history: strong opioid	73 (48.3)	7 (36.8)	0.234
pain treatment history: weak opioid	67 (44.4)	11 (57.9)	0.273
complications category: none	37 (24.5)	2 (10.5)	0.374
complications category: one	51 (33.8)	9 (47.4)	0.280
complications category: three or more	15 (9.9)	2 (10.5)	0.020
complications category: two	48 (31.8)	6 (31.6)	0.004

Note. Completeness requires the recorded QLQ-C15-PAL global and ESAS total scores at T0, T1, and T2; FAMCARE was not included.

Supplementary Table S4. Full balance diagnostics before and after matching.

Variable	Level	SMD before	SMD after	Absolute SMD after
age	continuous	-0.184	-0.074	0.074
BMI	continuous	-0.013	-0.027	0.027
KPS	continuous	0.262	-0.007	0.007
QLQ global T0	continuous	-0.067	-0.065	0.065
ESAS total T0	continuous	-0.086	-0.039	0.039
sex	female	0.016	0.072	0.072
sex	male	-0.016	-0.072	0.072
education	1	-0.102	0.000	0.000
education	2	0.042	-0.071	0.071
education	3	0.019	0.059	0.059
education	4	0.056	0.041	0.041
monthly income	1	-0.142	-0.048	0.048
monthly income	2	-0.059	0.025	0.025
monthly income	3	0.254	0.029	0.029
marital status	divorced or single	-0.075	0.000	0.000
marital status	married	0.119	-0.053	0.053
marital status	widowed	-0.081	0.058	0.058
caregiver relationship	child	0.010	0.047	0.047
caregiver relationship	none or other	0.170	0.000	0.000
caregiver relationship	other relative	0.056	0.048	0.048
caregiver relationship	spouse	-0.089	-0.071	0.071
cancer stage	III	0.150	-0.065	0.065
cancer stage	IV	-0.150	0.065	0.065
diagnosis category	gastrointestinal	0.084	0.025	0.025
diagnosis category	genitourinary	0.062	-0.039	0.039
diagnosis category	gynecologic breast	0.093	0.000	0.000
diagnosis category	other	-0.026	0.041	0.041
diagnosis category	respiratory	-0.190	-0.027	0.027
pain treatment history	none or nonopioid	-0.175	0.092	0.092
pain treatment history	strong opioid	0.026	0.000	0.000
pain treatment history	weak opioid	0.079	-0.047	0.047
complications category	none	0.098	0.084	0.084
complications category	one	0.034	-0.049	0.049
complications category	three or more	-0.017	-0.039	0.039
complications category	two	-0.109	0.000	0.000

Supplementary Table S5. Candidate patients retained versus not retained after matching.

Characteristic	Matched (n=170)	Unmatched (n=54)	Absolute SMD
Age, years	66.92±9.49	68.35±9.33	0.152
BMI, kg/m²	21.24±2.96	21.91±2.52	0.245
KPS	59.32±8.25	57.50±12.12	0.176
QLQ-C15-PAL global at T0	67.48±6.13	67.66±6.87	0.029
ESAS total at T0	32.25±5.02	32.65±4.67	0.081
sex: female	71 (41.8)	26 (48.1)	0.129
sex: male	99 (58.2)	28 (51.9)	0.129
education: 1	40 (23.5)	17 (31.5)	0.179
education: 2	81 (47.6)	19 (35.2)	0.255
education: 3	34 (20.0)	14 (25.9)	0.141
education: 4	15 (8.8)	4 (7.4)	0.052
monthly income: 1	72 (42.4)	28 (51.9)	0.191
monthly income: 2	61 (35.9)	22 (40.7)	0.100
monthly income: 3	37 (21.8)	4 (7.4)	0.415
marital status: divorced or single	10 (5.9)	9 (16.7)	0.346
marital status: married	124 (72.9)	30 (55.6)	0.369
marital status: widowed	36 (21.2)	15 (27.8)	0.154
caregiver relationship: child	82 (48.2)	20 (37.0)	0.228
caregiver relationship: none or other	2 (1.2)	2 (3.7)	0.164
caregiver relationship: other relative	11 (6.5)	8 (14.8)	0.273
caregiver relationship: spouse	75 (44.1)	24 (44.4)	0.007
cancer stage: III	26 (15.3)	9 (16.7)	0.037
cancer stage: IV	144 (84.7)	45 (83.3)	0.037
diagnosis category: gastrointestinal	59 (34.7)	13 (24.1)	0.235
diagnosis category: genitourinary	17 (10.0)	4 (7.4)	0.092
diagnosis category: gynecologic breast	34 (20.0)	8 (14.8)	0.137
diagnosis category: other	15 (8.8)	7 (13.0)	0.133
diagnosis category: respiratory	45 (26.5)	22 (40.7)	0.306
pain treatment history: none or nonopioid	12 (7.1)	11 (20.4)	0.394
pain treatment history: strong opioid	80 (47.1)	28 (51.9)	0.096
pain treatment history: weak opioid	78 (45.9)	15 (27.8)	0.382
complications category: none	39 (22.9)	14 (25.9)	0.070
complications category: one	60 (35.3)	8 (14.8)	0.486
complications category: three or more	17 (10.0)	7 (13.0)	0.093
complications category: two	54 (31.8)	25 (46.3)	0.301

Supplementary Table S6. Post-matching covariate-adjusted models.

Outcome	Time	Difference (95% CI)	P
QLQ_global	T0	-0.60 (-2.76 to 1.56)	0.586
QLQ_global	T1	10.69 (8.52 to 12.86)	<0.001
QLQ_global	T2	16.56 (14.36 to 18.76)	<0.001
ESAS_total	T0	-0.23 (-1.91 to 1.46)	0.790
ESAS_total	T1	-9.96 (-11.66 to -8.27)	<0.001
ESAS_total	T2	-17.82 (-19.54 to -16.10)	<0.001

Note. Models adjusted for age, sex, KPS, diagnosis, marital status, and caregiver relationship; difference is internet minus conventional.

Supplementary Table S7. Patient-only random-intercept sensitivity models.

Outcome	Time	Difference (95% CI)	P
QLQ_global	T0	-0.40 (-2.68 to 1.88)	0.732
QLQ_global	T1	10.88 (8.59 to 13.17)	<0.001
QLQ_global	T2	16.75 (14.43 to 19.07)	<0.001
ESAS_total	T0	-0.20 (-1.95 to 1.56)	0.825
ESAS_total	T1	-9.94 (-11.70 to -8.17)	<0.001
ESAS_total	T2	-17.78 (-19.56 to -15.99)	<0.001

Note. The primary model included matched-pair and patient random intercepts; this sensitivity model omitted the pair intercept.

Supplementary Table S8. Explicit baseline-outcome-adjusted follow-up models.

Outcome	Time	Difference (95% CI)	P
QLQ_global	T1	11.30 (9.85 to 12.74)	<0.001
QLQ_global	T2	17.15 (15.66 to 18.65)	<0.001
ESAS_total	T1	-9.77 (-10.84 to -8.70)	<0.001
ESAS_total	T2	-17.55 (-18.66 to -16.44)	<0.001

Note. Models at T1/T2 adjusted for the corresponding T0 recorded score because within-day initial assignment/T0 ordering was indeterminate.

Supplementary Table S9. Inverse-probability-of-observation weighted models.

Outcome	Time	Difference (95% CI)	P
QLQ_global	T0	-0.40 (-2.55 to 1.75)	0.717
QLQ_global	T1	10.87 (8.71 to 13.04)	<0.001
QLQ_global	T2	16.75 (14.55 to 18.95)	<0.001
ESAS_total	T0	-0.20 (-1.91 to 1.51)	0.821
ESAS_total	T1	-9.94 (-11.66 to -8.22)	<0.001
ESAS_total	T2	-17.76 (-19.50 to -16.01)	<0.001

Note. Stabilized weights were truncated at the 1st and 99th percentiles. Observation models included baseline and time-varying completion predictors.

Supplementary Table S9A. Weight diagnostics.

Outcome	1% truncation	99% truncation	Raw min	Raw median	Raw max
QLQ_global	0.920	1.192	0.889	0.996	1.303
ESAS_total	0.919	1.154	0.889	0.996	1.344

Supplementary Table S10. Differential pattern-mixture stress test.

Outcome	Scenario	Delta for missing internet values	Time	Difference (95% CI)	P
QLQ_global	MAR prediction	0	T0	-0.40 (-2.50 to 1.71)	0.711
QLQ_global	MAR prediction	0	T1	10.90 (8.79 to 13.00)	<0.001
QLQ_global	MAR prediction	0	T2	17.09 (14.98 to 19.19)	<0.001
QLQ_global	Moderate differential MNAR	-5	T0	-0.40 (-2.50 to 1.71)	0.711
QLQ_global	Moderate differential MNAR	-5	T1	10.78 (8.67 to 12.89)	<0.001
QLQ_global	Moderate differential MNAR	-5	T2	16.85 (14.75 to 18.96)	<0.001
QLQ_global	Severe differential MNAR	-10	T0	-0.40 (-2.52 to 1.72)	0.713
QLQ_global	Severe differential MNAR	-10	T1	10.66 (8.54 to 12.78)	<0.001
QLQ_global	Severe differential MNAR	-10	T2	16.62 (14.50 to 18.74)	<0.001
ESAS_total	MAR prediction	0	T0	-0.20 (-1.89 to 1.49)	0.819
ESAS_total	MAR prediction	0	T1	-10.08 (-11.77 to -8.38)	<0.001
ESAS_total	MAR prediction	0	T2	-17.60 (-19.29 to -15.90)	<0.001
ESAS_total	Moderate differential MNAR	3	T0	-0.20 (-1.89 to 1.50)	0.819
ESAS_total	Moderate differential MNAR	3	T1	-10.01 (-11.70 to -8.31)	<0.001
ESAS_total	Moderate differential MNAR	3	T2	-17.46 (-19.15 to -15.76)	<0.001
ESAS_total	Severe differential MNAR	6	T0	-0.20 (-1.90 to 1.51)	0.820
ESAS_total	Severe differential MNAR	6	T1	-9.93 (-11.64 to -8.23)	<0.001
ESAS_total	Severe differential MNAR	6	T2	-17.31 (-19.02 to -15.61)	<0.001

Note. Missing conventional-care outcomes were set to fixed-effect MAR predictions. Missing internet-care values were shifted unfavorably by −5/−10 for QLQ global and +3/+6 for ESAS total. This is a deterministic stress test, not multiple imputation.

Supplementary Table S11. Strict target-window sensitivity analysis.

Outcome	Time	Difference (95% CI)	P
QLQ_global	T0	-0.40 (-2.55 to 1.75)	0.717
QLQ_global	T1	10.77 (8.60 to 12.94)	<0.001
QLQ_global	T2	16.91 (14.68 to 19.14)	<0.001
ESAS_total	T0	-0.20 (-1.90 to 1.50)	0.820
ESAS_total	T1	-9.80 (-11.51 to -8.08)	<0.001
ESAS_total	T2	-17.95 (-19.71 to -16.19)	<0.001

Note. T1 was restricted to 21–35 days and T2 to 49–63 days.

Supplementary Table S11A. Records inside and outside target windows.

Group	Time	Total	Within window	Outside window
conventional	T1	85	83	2
conventional	T2	85	79	6
internet	T1	85	84	1
internet	T2	85	81	4

Supplementary Table S12. Descriptive summary of the exploratory recorded care-satisfaction measure.

Group	Time	Observed n	Missing n	Mean±SD	Median [IQR]
conventional	T0	48	37	51.42±4.25	51.45 [49.00–54.45]
conventional	T1	85	0	52.63±5.75	52.30 [48.50–56.00]
conventional	T2	85	0	50.60±6.19	51.50 [46.50–55.00]
internet	T0	51	34	52.48±3.38	52.90 [49.55–55.20]
internet	T1	85	0	65.27±4.41	65.40 [61.80–68.30]
internet	T2	85	0	66.98±4.85	66.60 [63.60–70.50]

Note. The archived field was labeled FAMCARE_total, but version, respondent identity, respondent continuity, administration mode, and scoring workflow could not be verified. No confirmatory inference was performed; reference 17 in the manuscript supports FAMCARE rather than QLQ-C15-PAL scoring.

Supplementary Table S13. Supportive outcomes with FDR correction.

Outcome	Time	Difference (95% CI)	Nominal P	FDR-adjusted P
QLQ_physical	T1	11.66 (8.39 to 14.94)	<0.001	<0.001
QLQ_physical	T2	17.24 (13.86 to 20.62)	<0.001	<0.001
QLQ_pain	T1	-10.24 (-12.78 to -7.70)	<0.001	<0.001
QLQ_pain	T2	-12.77 (-15.40 to -10.14)	<0.001	<0.001
QLQ_fatigue	T1	-7.65 (-10.21 to -5.09)	<0.001	<0.001
QLQ_fatigue	T2	-8.61 (-11.26 to -5.95)	<0.001	<0.001

Supplementary Table S14. Exploratory T3 analysis.

Outcome	Time	Difference (95% CI)	P
QLQ_global	T0	-0.40 (-2.59 to 1.80)	0.722
QLQ_global	T1	10.88 (8.67 to 13.09)	<0.001
QLQ_global	T2	16.77 (14.52 to 19.02)	<0.001
QLQ_global	T3	18.38 (16.09 to 20.67)	<0.001
ESAS_total	T0	-0.20 (-2.01 to 1.61)	0.831
ESAS_total	T1	-9.90 (-11.73 to -8.08)	<0.001
ESAS_total	T2	-17.71 (-19.57 to -15.86)	<0.001
ESAS_total	T3	-22.29 (-24.16 to -20.41)	<0.001

Note. T3 was a heterogeneous withdrawal/final assessment. Estimates are vulnerable to timing heterogeneity, survivor selection, and unavailable exact death dates and are not confirmatory.

Supplementary Table S15. Conventional 1:1 nearest-neighbor propensity-score matching sensitivity analysis.

Recorded outcome	Time	Matched pairs	Difference (95% CI)	P value
QLQ-C15-PAL global quality-of-life score	T1	88	10.62 (8.48 to 12.76)	<0.001
QLQ-C15-PAL global quality-of-life score	T2	88	15.70 (13.53 to 17.87)	<0.001
ESAS total symptom-burden score	T1	88	−9.14 (−10.73 to −7.54)	<0.001
ESAS total symptom-burden score	T2	88	−17.25 (−18.87 to −15.63)	<0.001

Note. Difference is initial internet-pathway assignment minus conventional-pathway assignment. Matching used the same propensity-score model, common support, a 0.20-SD logit-propensity caliper, 1:1 nearest available control without replacement, deterministic high-to-low treated ordering, patient-ID tie breaking, and no auxiliary covariate-distance term. The maximum post-match absolute SMD was 0.163. Absolute SMDs >0.10 remained for monthly income level 2 (0.163), baseline QLQ-C15-PAL global score (0.148), widowed status (0.143), no/nonopioid pain-treatment history (0.141), respiratory diagnosis (0.127), three-or-more complications (0.110), no complications (0.107), and married status (0.104). Effects were directionally consistent and slightly attenuated relative to the primary pragmatic composite-distance match; because balance was weaker, this sensitivity analysis supports direction only and does not supersede the primary analysis.

Supplementary Figures

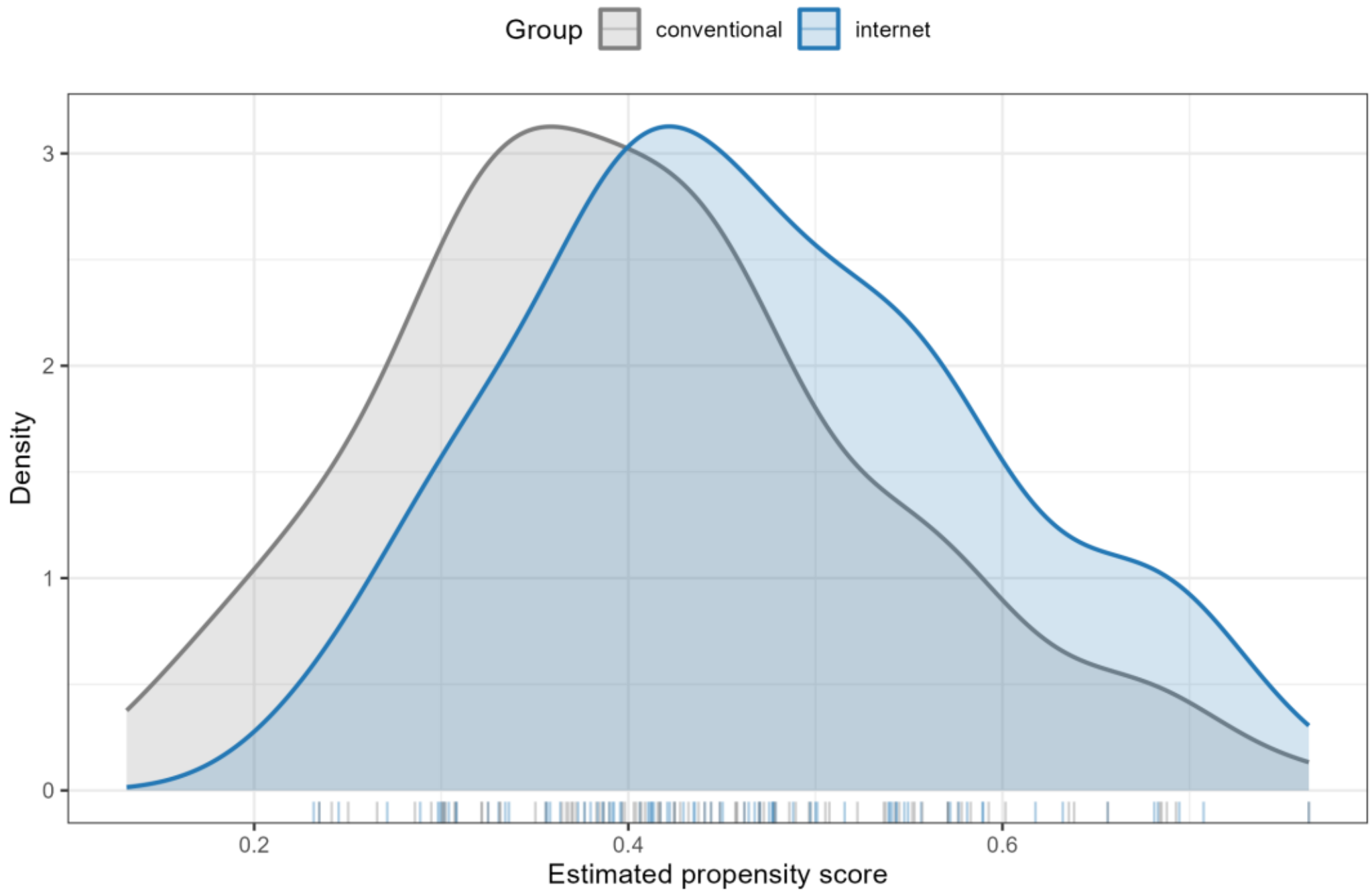

Supplementary Figure S1. Propensity-score distributions in the candidate cohort; rugs denote matched patients.

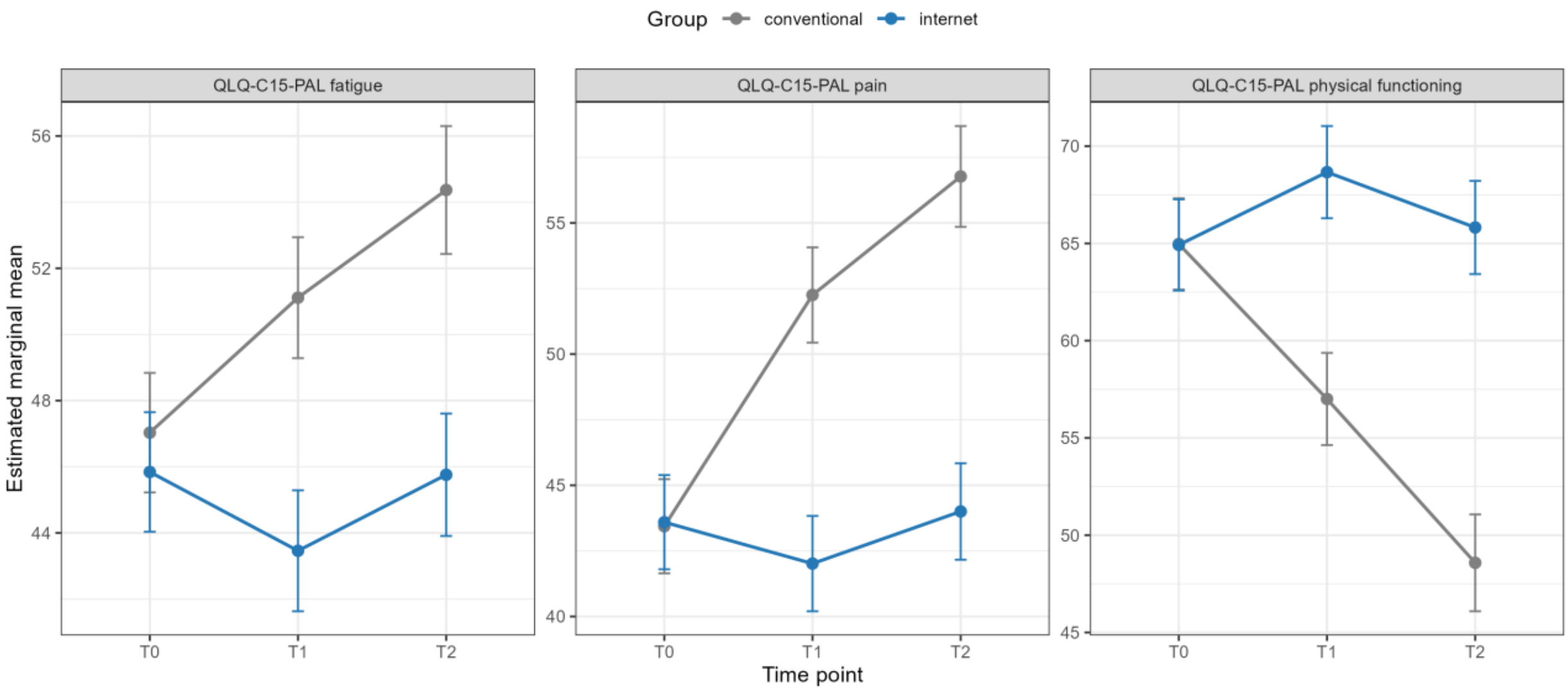

Supplementary Figure S2. Estimated marginal means and 95% confidence intervals for supportive recorded QLQ-C15-PAL outcome scores.

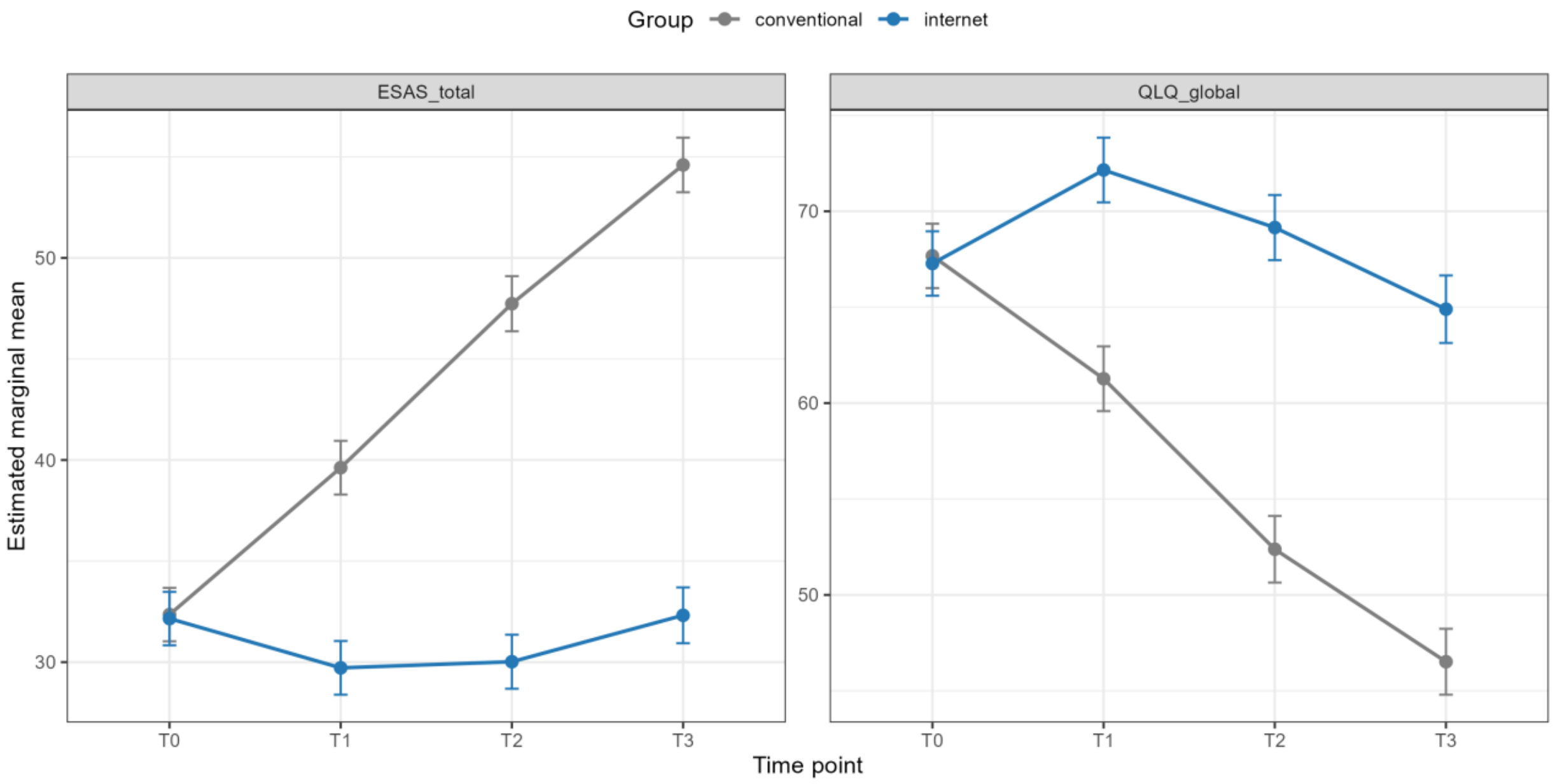

Supplementary Figure S3. Exploratory recorded T0–T3 score trajectories. T3 was not a scheduled endpoint, and exact death dates were unavailable.
